# Comparing current and emerging practice models for the extrapolation of survival data: a simulation study and case-study

**DOI:** 10.1186/s12874-021-01460-1

**Published:** 2021-11-27

**Authors:** Benjamin Kearns, Matt D. Stevenson, Kostas Triantafyllopoulos, Andrea Manca

**Affiliations:** 1 School of Health and Related Research. Regent Court (ScHARR), The University of Sheffield, 30 Regent Street, Sheffield, S1 4DA UK; 2 School of Mathematics and Statistics, The University of Sheffield, 30 Regent Street, Sheffield, S1 4DA UK; 3 Centre for Health Economics, The University of York, York, UK

**Keywords:** Survival analysis, Forecasting, Extrapolation

## Abstract

**Background:**

Estimates of future survival can be a key evidence source when deciding if a medical treatment should be funded. Current practice is to use standard parametric models for generating extrapolations. Several emerging, more flexible, survival models are available which can provide improved within-sample fit. This study aimed to assess if these emerging practice models also provided improved extrapolations.

**Methods:**

Both a simulation study and a case-study were used to assess the goodness of fit of five classes of survival model. These were: current practice models, Royston Parmar models (RPMs), Fractional polynomials (FPs), Generalised additive models (GAMs), and Dynamic survival models (DSMs). The simulation study used a mixture-Weibull model as the data-generating mechanism with varying lengths of follow-up and sample sizes. The case-study was long-term follow-up of a prostate cancer trial. For both studies, models were fit to an early data-cut of the data, and extrapolations compared to the known long-term follow-up.

**Results:**

The emerging practice models provided better within-sample fit than current practice models. For data-rich simulation scenarios (large sample sizes or long follow-up), the GAMs and DSMs provided improved extrapolations compared with current practice. Extrapolations from FPs were always very poor whilst those from RPMs were similar to current practice. With short follow-up all the models struggled to provide useful extrapolations. In the case-study all the models provided very similar estimates, but extrapolations were all poor as no model was able to capture a turning-point during the extrapolated period.

**Conclusions:**

Good within-sample fit does not guarantee good extrapolation performance. Both GAMs and DSMs may be considered as candidate extrapolation models in addition to current practice. Further research into when these flexible models are most useful, and the role of external evidence to improve extrapolations is required.

**Supplementary Information:**

The online version contains supplementary material available at 10.1186/s12874-021-01460-1.

## Background

Accurate extrapolations of future survival can be pivotal evidence sources for decision-makers when determining if a medical treatment should be funded. In England, the National Institute for Health and Care Excellence (NICE) – which provides national guidance on if treatments should be funded – requires that all relevant health benefits of a treatment be quantified. This is to enable consistent and fair decision making across diverse treatments. Hence if a treatment impacts on survival this should be extrapolated to provide estimates of lifetime survival benefit. Recent reviews of cancer treatments appraised by NICE found that between 2011 and 2017, every appraisal involved extrapolation [1]. On average, evidence on treatment effectiveness was available for 2.9 years, and extrapolated to 25.2 years [2].

Current practice is to use standard parametric survival models (such as the exponential and Weibull) when analysing and extrapolating survival data [1]. There is a growing awareness that such models may not be sufficiently flexible to accurately capture the complex hazard patterns that may arise in practice [3, 4]. There are several more-advanced survival models which may give improved fit to the observed data [5]. However, it is unclear if improved within-sample fit will lead to improved extrapolation performance, and there is dearth of comparative studies which include these flexible emerging practice models. The aim of this study was to compare both the within-sample goodness of fit and the extrapolation performance of current and emerging practice models. This was achieved using both a simulation study and a case-study. Use of a case-study demonstrates the performance of survival models using real data, whilst use of a simulation study avoids estimates of performance being driven by the quirks of a single dataset.

## Methods

This section begins with an overview of the survival models used, followed by details of the simulation study and case-study.

### Survival models

For brevity, the descriptions here focus on the qualitative properties of the models. Technical details may be found in the references provided. A key distinction between the models is if they are global, piecewise, or local. For global models, parameter estimates are the same at all time points (constant over time). For piecewise models, parameter estimates are constant over time within specified time intervals but allowed to vary across time intervals. For local models, parameter estimates vary over time. Five classes of survival model were considered:

1. Current practice [6, 7]. Eight standard parametric global survival models were considered: exponential, Weibull, Gompertz, log-logistic, lognormal, gamma, generalised gamma, and generalised F. The first five models may be written as linear models; this assumption of linearity is relaxed by the emerging practice models of the other four model classes.
2. Royston Parmar models (RPMs) [8, 9]. These extend linear models by the incorporation of piecewise cubic polynomials, which are restricted to have the same value at a set of `knots’, with the number of knots determining the complexity of the model. Up to five internal knots were considered, with two specifications which correspond to extensions of the Weibull and log-logistic models, respectively.
3. Fractional polynomials (FPs) [10, 11]. These are global models; one and two polynomial terms of the logarithm of time were considered, giving FP (1) and FP (2) models, respectively. Powers were taken from the set [− 2, − 1, − 0.5, 0, 0.5, 1, 2, 3], resulting in eight FP (1) and 36 FP (2) models. It is possible to choose between FP (1) and FP (2) models using a closed-test procedure [12], but for this study FP (1) and FP (2) models were kept separate. This is because FP (2) models are more complex than FP (1) models, and there was interest in seeing if extrapolation performance varied by model complexity.
4. Generalised additive models (GAMs) [13, 14]. The models considered start with a Weibull, and add complexity via additional parameters, known as bases. For this study regression splines were used, with a maximum dimension of ten. The likelihood for these models includes a term that penalises for model complexity, which leads to shrinkage of model parameters. GAMs are global models.
5. Dynamic survival models (DSMs) [5, 15]. These are local models which introduce flexibility by allowing model parameters to evolve over time, as described by a time-series. Two models were considered. Both extend a linear Weibull model by allowing the trend parameter to follow a random walk. The first model (‘local trend’) extrapolated this trend indefinitely. The second model (‘damped trend’) successively decreased the extrapolated trend as the time horizon increased so that eventually the trend became zero and extrapolations were constant. The degree of dampening was estimated from the data.

### Simulation study

The reporting of the simulation study follows published guidance [16]. Components of the simulation study are reported based on their aims (provided in the introduction), data generating mechanisms, methods (models), estimand, and performance measures.

#### Data generating mechanism

A two-component mixture-Weibull model was used; it may be interpreted as representing two sub-populations of patients with either a high hazard (short survival) or a low hazard (long survival) The survival and hazard functions are given by [17]:

$${\mathrm{S}}_{{\mathrm{t}}_{\mathrm{i}}}=\uprho \mathrm{exp}\left(-{\Lambda}_1{\mathrm{t}}_{\mathrm{i}}^{\upgamma_1}\right)+\left(1-\uprho \right)\exp \left(-{\Lambda}_2{\mathrm{t}}_{\mathrm{i}}^{\upgamma_2}\right)$$

$${\uplambda}_{{\mathrm{t}}_{\mathrm{i}}}=\frac{\Lambda_1{\upgamma}_1{\mathrm{t}}_{\mathrm{i}}^{\upgamma_1^{-1}}\uprho \mathrm{exp}\left(-{\Lambda}_1{\mathrm{t}}_{\mathrm{i}}^{\upgamma_1}\right)+{\Lambda}_2{\upgamma}_2{\mathrm{t}}_{\mathrm{i}}^{\upgamma_2^{-1}}\left(1-\uprho \right)\exp \left(-{\Lambda}_2{\mathrm{t}}^{\upgamma_2}\right)}{{\mathrm{S}}_{{\mathrm{t}}_{\mathrm{i}}}}$$

respectively, where γ and Λ are the respective shape and scale parameters (indexed by component), and ρ is the mixing proportion. The values used are: γ_1_ = 1.8, Λ_1_ = 0.02, γ_2_ = 1.4, Λ_2_ = 2.3, and ρ = 0.5. This was designed to reflect a ‘true’ hazard with two turning points (at approximately 0.5 and 1.75 years), and a long-term increasing hazard (reflecting the impact of ageing).

Nine scenarios were simulated, with 200 datasets simulated for each scenario. These scenarios corresponded to three different sample sizes (small = 100, medium = 300, large = 600), and three different lengths of follow-up (short = 2 years, medium = 3 years, long = 4 years). Hence all scenarios included both turning points in the hazard function but varied by how soon after the last turning point follow-up ended. The sample sizes were chosen to be representative of those typically seen in clinical practice at the point of reimbursement decision making. Details on these scenarios are provided in Table 1 and visualised in Fig. A1 in Additional file 1.

**Table 1:** Details of the nine scenarios simulated

Scenario	Follow-up (survival %)	Sample size
Short follow-up, small sample size	2 years (46.8%)	100
Short follow-up, medium sample size		300
Short follow-up, large sample size		600
Medium follow-up, small sample size	3 years (43.3%)	100
Medium follow-up, medium sample size		300
Medium follow-up, large sample size		600
Long follow-up, small sample size	4 years (39.2%)	100
Long follow-up, medium sample size		300
Long follow-up, large sample size		600

#### Methods

The five classes of survival model previously described were included. For current practice, the generalised F was not included due to a lack of convergence. Further, the main results do not include the Gompertz model due its very poor extrapolation performance. Results including the Gompertz are provided in Additional file 1. For the two DSM specifications (local trend, damped trend), a constant level (intercept) as well as a time-varying local intercept was considered, resulting in four DSMs. For the first three model classes multiple specifications are possible. In practice, the choice between these specifications would be based on a combination of clinical considerations and empirical goodness of fit. For this study, the choice between model specifications was based solely on Akaike information criteria (AIC) for current practice, RPM, FP [1] and FP [2] models [18]. All analyses were performed in R, using a variety of packages [7, 19, 20]. Full details on the packages used and implementation and provided in Additional file 2.

#### Estimand and performance measures

The estimand was the mean of the natural logarithm of the time-varying hazard function *λ*_*t*_. The primary performance measure used was the mean (of the) squared error (MSE), with bias as a secondary performance measure. For MSE smaller values indicate better model performance, for bias this is indicated by values closer to zero. Further details on the justification for these measures and their definition are provided in Additional file 1.

### Case-study

Patient-level data were obtained for the clinical trial COU-AA-301 (NCT00638690) from the Yale University Open Data Access Project [21]. This trial compared abiraterone acetate (henceforth referred to as abiraterone) to placebo in people with castration-resistant prostate cancer previously treated with docetaxel-based chemotherapy. The available data was for 1183 people (abiraterone = 791, placebo = 392) with almost complete follow-up: median 36.2 months, by which time 984 (82.3%) people had died. An early cut of the data has been published, based on a median follow-up of 12.8 months and 552 deaths (46.2%) [22]. The five classes of survival model were applied to the early cut of the data, with the more complete data used to evaluate the extrapolation performance. More details on the available data, including how the early cut was replicated, are provided in Additional file 1. For classes one to three, multiple models may be fit. The choice of model(s) to use for extrapolations was based on a combination of the plausibility of extrapolations and the goodness of fit to the observed data, quantified by both the AIC and Bayesian information criteria (BIC) [18].

## Results

### Simulation study

**Fig. 1:**
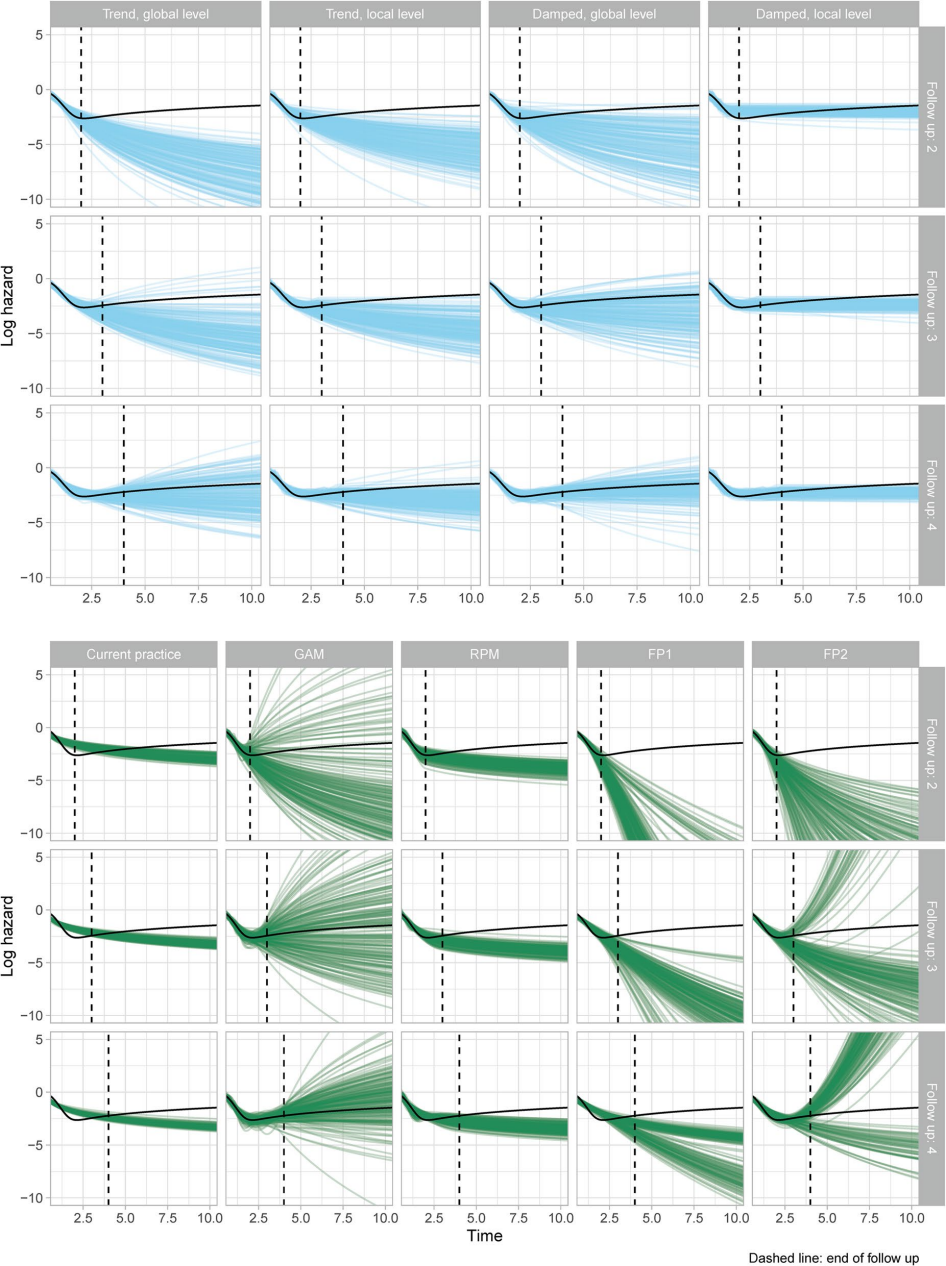
Model estimates of the log-hazard (blue lines) and true values (black lines)

**Fig. 2:**
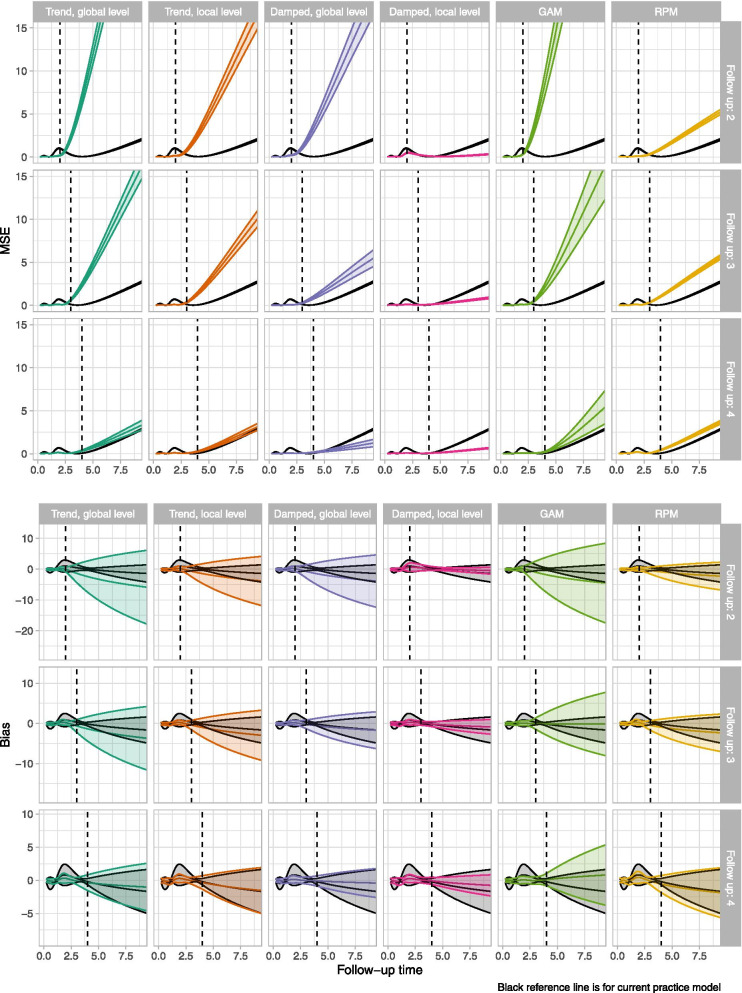
Mean squared error and bias values by time (within-sample and extrapolations)

For each model, the visual patterns of within-sample fit and extrapolations were broadly similar across the nine scenarios considered. Increasing the sample size led to a reduction in the variation of extrapolations as expected but had little other effect. Results were more sensitive to changes in length of follow-up. For a sample size of 300 and all three follow-ups and all nine models, Fig. 1 demonstrates visual fit whilst Fig. 2 provides time- varying estimates of the MSE and bias (due to their very poor fit, FPs are excluded from Fig. 2). Plots for the remaining scenarios are provided in Additional file 1 (Figs. A2 to A5).

For the within-sample period, the current practice models provided a poor fit to the observed data for all the scenarios: the hazard was under-estimated for the first year and over-estimated for subsequent years, with neither turning-point in the hazard captured. The remaining models all provided visually improved within-sample fit, although they also typically had more variability in their estimates. With the shortest follow-up (2 years) none of the models identified the long-term increasing trend in the hazard function. With the longest follow-up (4 years) two dynamic models (local trend and damped trend models; both with a global level) along with the GAM identified the long-term increasing hazard; the remaining models did not. For the three models that identified the long-term increasing hazard, the bias in the extrapolations decreased with increasing sample-size; for the largest sample size they provided approximately unbiased estimates. The extrapolation performance of the two DSMs with a local level also improved with increasing sample size, but they consistently under-estimated the true hazard. In contrast, for the current practice models and RPMs, the bias was not reduced by increasing sample size. Results for a follow-up of 3 years were similar to those for 4 years, but with more uncertainty in the extrapolations. This uncertainty led to some extreme departures from the true hazard values for the GAMs and DSMs. In contrast, use of standard models or RPMs led to extrapolations that were always biased, but there were never any extreme departures from the truth.

In general, GAMs required less data (sample size or follow-up) than DSMs to identify the turning-point in the hazard, but GAMs also produced more variable extrapolation estimates than the DSMs. This large variation is a particular concern as in each appraisal (or analysis) only a single extrapolation would be obtained and there is a danger that it would correspond to one of the very poor extrapolations. For the DSMs, dampening the trend led to less variable extrapolations and lower average MSE and bias than the corresponding local trend models. Both FP model classes provided extremely poor extrapolations which very quickly tended towards zero or very large numbers and lacked face validity. Despite generally having the worst within-sample fit, current practice models often provided some of the best extrapolations with short-to-medium follow-up. However, as demonstrated in Fig. 1, the good extrapolation performance of the current practice models is an artifact of their poor within-sample fit, as the extrapolated (decreasing) hazards were by chance close to the true (increasing) hazards. Estimates from individual current practice models, including the Gompertz, are provided in Additional file 1. The GAMs and RPMs are both spline-based models but produced very different extrapolations. Further comparison of these models is provided in Additional file 1, which shows that GAMs were generally more complex than the RPMs.

Overall values of MSE and bias (averaged across the within- and out-of-sample time periods) are provided in Table 2. For each of the nine scenarios considered, the DSM with a damped trend and a local level provided the lowest MSE values. The next lowest MSE values were typically observed for the current practice and RPMs, despite these two model types predicting a long-term decrease in hazards for all nine scenarios. As the scenarios became more data rich (increasing follow-up and/or sample size), the performance of the DSMs improved relative to the other models. For example, with a sample size of 600 and four-years follow-up, the four DSMs had the lowest MSE of all the models considered. The class of FPs give the worst extrapolations for every scenario. This may be due to their sensitivity to extreme values, combined with extrapolating polynomial trends [23]. Omitting the FPs, the largest MSE values were observed for the GAM in seven of the nine scenarios. The poor performance of the GAMs is primarily driven by the large variability in extrapolations, as it provided the least-biased estimates in four scenarios. For the remaining five scenarios a DSM provided the least-biased estimates (two each for the two DSMs with a damped trend, one for the local trend global level DSM).

**Table 2:** Goodness of fit over the entire time horizon

Overall mean	Sample size: 100			Sample size: 300			Sample size: 600		
squared error	FU: 2 years	FU: 3 years	FU: 4 years	FU: 2 years	FU: 3 years	FU: 4 years	FU: 2 years	FU: 3 years	FU: 4 years
Damped trend, local level	0.51	0.34	0.42	0.23	0.38	0.29	0.26	0.41	0.27
Current practice	1.01	1.19	1.26	0.94	1.15	1.19	0.90	1.12	1.15
Royston-Parmar model	1.98	2.38	1.87	2.21	2.38	1.50	2.25	2.36	1.40
Damped trend, global level	3.75	4.98	2.36	7.88	2.29	0.52	8.07	1.41	0.35
Local trend, local level	3.33	4.41	2.96	6.86	4.13	1.26	9.18	3.39	0.71
Local trend, global level	6.03	7.12	4.27	15.61	6.67	1.36	18.04	4.65	0.57
Generalised additive model	32.89	18.16	6.85	18.49	6.59	2.12	20.27	4.09	1.53
Fractional polynomial: order 1	312.40	103.82	22.49	326.43	41.25	8.61	331.78	35.71	9.14
Fractional polynomial: order 2	531.90	258.30	147.35	205.23	55.05	85.21	121.62	24.07	65.57
**Overall bias**									
Damped trend, local level	0.38	−0.03	− 0.19	− 0.12	− 0.35	− 0.40	− 0.30	−0.30	− 0.31
Current practice	−0.36	− 0.37	− 0.35	− 0.55	−0.55	− 0.54	−0.60	− 0.58	−0.56
Royston-Parmar model	−0.92	−1.07	− 1.10	− 1.07	− 1.10	− 1.11	−0.88	− 0.79	−0.77
Damped trend, global level	−0.35	− 1.83	− 1.80	− 1.17	−0.78	− 0.56	−0.76	− 0.18	−0.14
Local trend, local level	−0.93	− 1.80	−2.14	− 1.32	−1.36	− 1.23	−1.06	− 0.64	− 0.48
Local trend, global level	− 1.36	− 2.85	−3.13	−1.73	− 1.72	−1.30	− 1.31	− 0.45	− 0.11
Generalised additive model	−1.55	−2.18	− 1.99	− 0.06	− 0.09	0.05	0.15	0.31	0.23
Fractional polynomial: order 1	−10.52	−11.87	− 12.24	−5.36	−4.10	− 3.91	−2.37	−1.75	− 1.83
Fractional polynomial: order 2	−5.45	−8.03	−6.72	1.45	− 0.79	−1.84	4.05	3.53	3.08

*FU* Follow-up

### Case-study

**Fig. 3:**
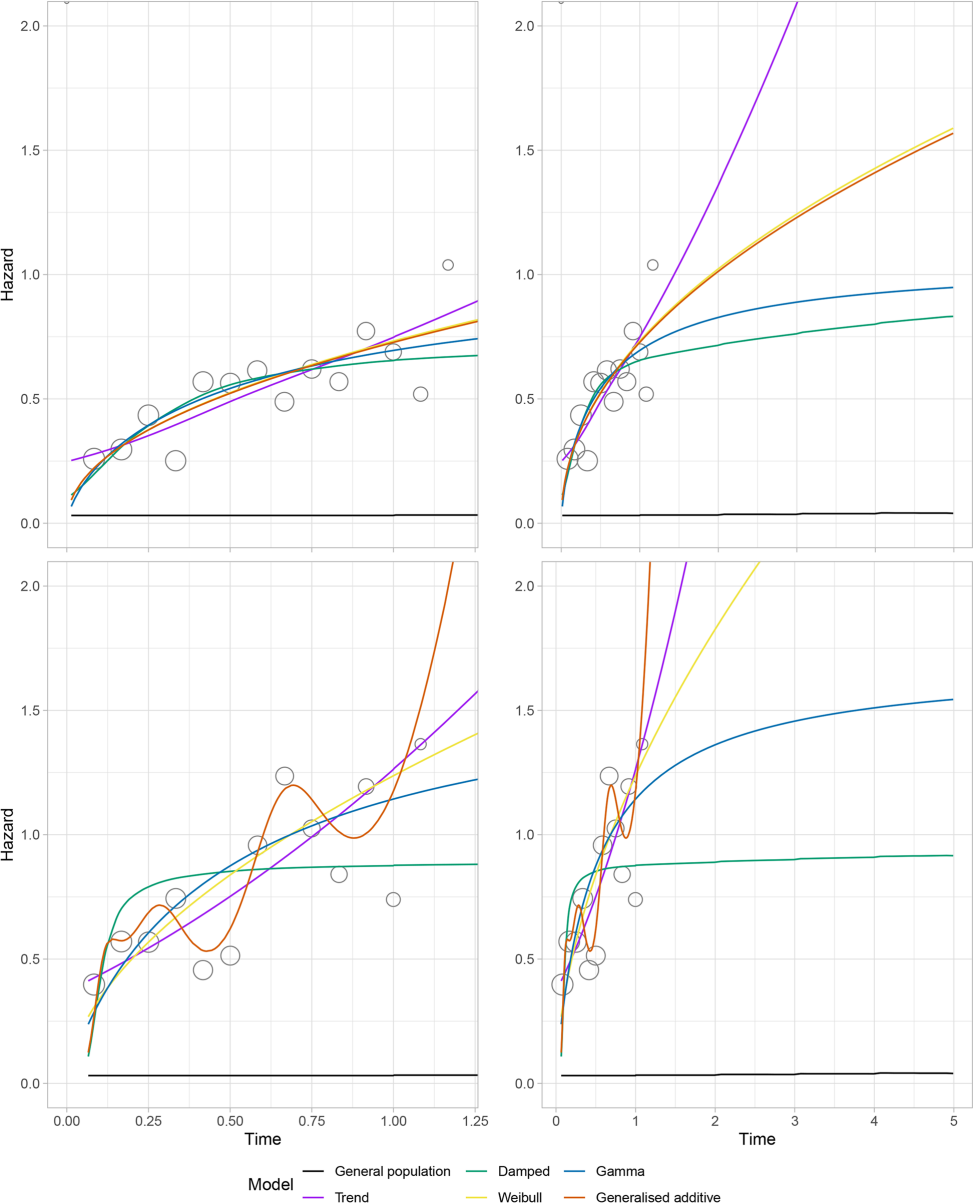
Within-sample fit and extrapolations from candidate extrapolation models

Within-sample estimates and extrapolations from the models selected for extrapolations are provided in Fig. 3, which also includes general population hazard values as a reference. Information criteria for the standard models and RPMs are provided in Additional file 1 (Table A4). The Weibull and gamma models had very similar AIC and BIC values and for both treatment groups were the two best standard models. Hence both were considered for extrapolation. For the abiraterone group, the RPM corresponding to the Weibull had the lowest AIC and BIC; models with increasing complexity had decreasing within-sample fit. For the placebo group use of AIC and BIC led to contrary findings. The BIC supported the use of a Weibull, whilst more complex models had better AIC values. Visually the more complex models appeared to be over-fitting the data, suggesting that in this instance AIC may not be sufficiently penalising model complexity. Since the best-fitting (and plausible) RPM was the Weibull for both groups and this was already chosen as a standard model, no RPMs were used for extrapolation. For both groups, the FP1 with the lowest AIC was the same model as a Weibull. This model provided very similar visual estimates (within-sample and extrapolations) to the best-fitting FP2 but had lower AIC. As such, the FP1 model corresponding to the Weibull was chosen for both groups.

The fitted GAM provided visual estimates that were very similar to the RPM with the lowest AIC, suggesting a Weibull for abiraterone and a function with six turning points in the hazard for the placebo group. These are used for extrapolation even though it is noted that the placebo GAM may be over-fitting the data (resulting in very large extrapolated hazards). For the abiraterone group, both DSMs provide similar estimates to the Weibull model up to about 9 months. After this time, the local trend model estimates higher hazards than the Weibull and the damped trend model estimates lower hazards. Similar extrapolations were observed for the placebo group, with the damped trend providing the lowest extrapolated hazards of all models considered and the local trend the second highest (below the GAM).

**Fig. 4:**
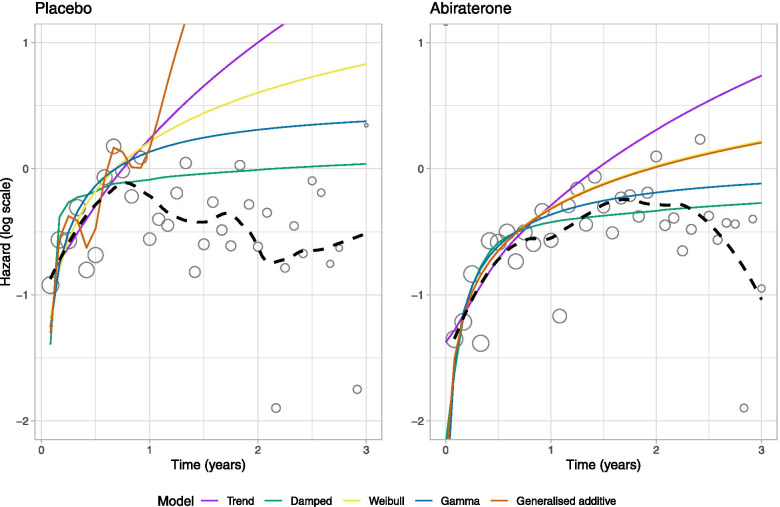
Comparisons of extrapolations against longer follow-up (dashed-lines)

A visual comparison of the model-estimates to the longer-term data is provided in Fig. 4, which also include a smooth non-parametric estimate (black-dashed line). For both treatment groups, the trend observed in the early data cut does not persist in the long-term. For the placebo group, the short-term increase in the hazard during the period of the interim data is followed by an almost immediate decrease. As such, none of the models provide good extrapolations. For the abiraterone group, the hazard continues increasing to about 2.5 years albeit at a lower rate than was observed in the early cut. The damped trend model provides adequate extrapolations up to about 2.5 years. After this time, the observed hazards decrease, and none of the models provide a good description. Extrapolations beyond 3 years were not considered due to the small sample sizes (at 3 years the number of patients remaining in the study was 62 and 23 for the abiraterone and placebo arms, respectively, whilst at 3.5 years the numbers were 24 and 3, respectively).

## Discussion

The within-sample fit and extrapolation performance of several survival models was evaluated in nine simulated scenarios covering different lengths of follow up and different sample sizes. A single data-generating mechanism was used, with two turning points in the hazard function. Only the global-level DSMs and GAMs were able to correctly extrapolate an increasing hazard function, but only in the more data-rich scenarios, and extrapolations were highly variable.

Current practice models provided the worst within-sample estimates of all the models considered in the simulation study. The DSMs and emerging practice models were able to provide improved within-sample fit due to their increased flexibility. However, this extra flexibility sometimes resulted in overfitting and extrapolating short term trends in the data that were not present in the longer term. A stark example of this was observed for the two FP model classes, for which extrapolations tended sharply towards implausibly small or large values. The danger of the more flexible models overfitting was in general reduced with increased sample size or follow-up, which led to improved extrapolation performance. A corresponding improvement in extrapolation performance for the more data-rich scenarios was not observed for current practice models.

A strength of the simulation study is the large number of survival models considered. For each scenario DSMs, current practice, spline-based models, and fractional polynomials were all evaluated. When including different model specifications, collectively 62 different models were fit for each scenario, with nine models retained for estimating extrapolation performance. The use of model selection also showed that within-sample goodness of fit plays a very limited role in identifying models that provide accurate extrapolations. For example, the current practice model with the best within-sample fit typically provided the worst extrapolations. A further strength of the study is the novel use of time-varying estimands instead of a single summary measure of accuracy such as the estimate of lifetime mean survival, which is affected by both within and out-of sample fit (an accurate estimate may occur if short-term over-estimates of hazard and long-term under-estimates cancel out, or vice-versa).

There are limitations to the simulation study. Only a single data generating mechanism (a mixture Weibull) was considered, with only one set of parameters. The within-sample fit and extrapolation performance of the candidate models in other settings is currently unknown and would be a fruitful area for future research. The existing data generating mechanism included two turning points, so in this sense favoured the more flexible models. However, survival data are inherently complex with a multitude of potential competing effects, such as ageing, frailty, treatment benefits, and adverse events. Collectively these are likely to cause complex shapes in the hazard function. Cure models may also be used for survival data with a turning-point [24]. They were not used here as the simulation study did not involve a cured fraction nor was there any indication that the case-study included one. Future research could explore the performance of cure models under misspecification.

In the case-study, for both treatment groups the hazards observed in the early data cut were increasing and use of current practice models favoured either the Weibull or gamma. These both provided monotonically increasing extrapolated hazards. Similar extrapolations were obtained from the more flexible FPs, RPMs and GAMs, along with the local trend model. In contrast, the damped trend model provided extrapolations that increased at a much lower rate for both groups. For both treatment groups the true long-term hazards eventually decreased. As none of the considered models were able to extrapolate a turning point, their predictions were generally all poor. The damped trend model assumes that the hazard function will eventually change from increasing to constant; this is closest to what occurred in the full dataset. However, as this is a single case-study, the generalisability of this finding to other scenarios is unclear. This case-study emphasises that any extrapolations are only as good as the dataset that is used. If the unobserved future contains turning points, then any extrapolation model would do poorly unless it incorporates external data to identify the turning points.

The simulation study was relatively simple, comprising two monotonic (Weibull) hazard functions. Yet producing accurate extrapolations was challenging, even with a follow-up of 3 years. The dataset and results of this manuscript will provide useful test-cases and benchmarks for future research to see if it is possible to provide improved extrapolations. Collectively, the simulation study and case-study suggest several areas for future research. Future studies could seek to identify if there are certain situations when one or more of the model classes out-performs the other models, and so may be used as the default approach. The current results suggest that whilst use of a damped-trend DSM may be beneficial, there is a danger that it will provide worse extrapolations than current practice models, especially in data-poor scenarios. This motivates consideration of a variety of different models, with model choice made on a case-by-case basis. This choice would consider the specifics of the extrapolation problem, such as the plausibility of extrapolations, the richness of the available data, and the qualitative differences in extrapolations arising from different models. The results currently suggest that simpler models may be appropriate in data-poor settings, although there is a danger that no extrapolations will be useful in these situations. Further research is required to understand the conditions under which evidence are rich enough to justify a more complex model. As an alternative to model selection, model averaging could be performed [25]. The case-study demonstrated that extrapolations were poor when the available follow-up did not include all the turning-points in the hazard function. The simulation study showed that even if all the turning points are included extrapolations may still be poor. This illustrates the potential importance of incorporating external evidence to improve extrapolations [26]. Neither the case-study nor the simulation study explored the impact on extrapolations of including covariates effects. In general incorporating additional information is expected to improve within-sample fit, but is unlikely to alter the conclusions of this manuscript.

The simulation study demonstrated that in situations when survival outcomes may arise from distinct patient populations, current practice models are unlikely to provide accurate estimates of the observed data or realistic extrapolations. Of the emerging practice models considered, DSMs and GAMs were the only ones able to capture the long-term behaviour of the hazard function. However, extrapolations from these more flexible models were more variable than extrapolations from current practice models and had the potential to be less accurate. In the case-study neither the current nor emerging practice models were able to provide accurate extrapolations. To conclude, emerging practice models may be currently viewed as another option in the toolkit of methods for the analysis and extrapolation of survival data. More experience of these models when used with different datasets is required to provide more specific guidance about their role, including the situations when they are likely to be the most useful.

## Supplementary Information

**Additional file 1.**

**Additional file 2.**

## Data Availability

Code is provided to replicate the generation and analysis of the simulation study (Additional File 2). Data used for the case-study may be obtained upon request from the Yale University Open Data Access Project (https://yoda.yale.edu/how-request-data).
