# Supplementary material for: Comparing current and emerging practice models for the extrapolation of survival data: a simulation study and case-study
Source: BMC Med Res Methodol. 2021 Nov 27;21:263. doi: 10.1186/s12874-021-01460-1 (PMC8627632; doi:10.1186/s12874-021-01460-1)
Supplement: Supplementary file 1 — Additional file 1. [file 12874_2021_1460_MOESM1_ESM.docx]

## Simulation study methods: further details

Figure A1 shows the simulated hazards (in grey) for each scenario along with the true hazard (in black, which is the same for each scenario). Hence, for each scenario Figure A1 displays 200 grey lines (empirical hazard functions), where the number of observations for each hazard function is equal to the sample size for that scenario.

**Figure A1: Simulated hazards (grey-lines) for the nine scenarios, along with the truth (blackline)**


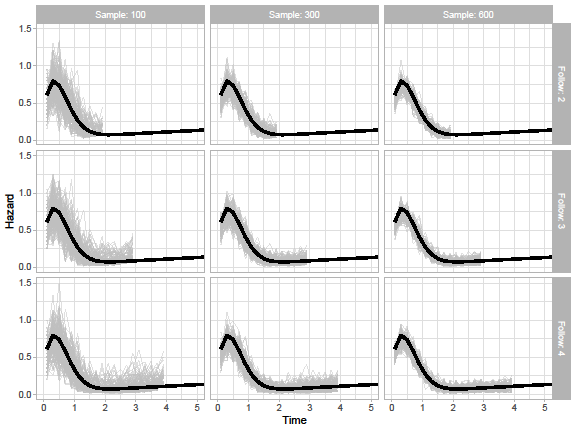


The estimand was the mean natural logarithm of the time-varying hazard function $\lambda_{t_{i}}$. The natural logarithm was used. This maps $\lambda_{t_{i}}$ to the range $\left( -\infty,\infty\right)$ and it may be assumed to be approximately Normally distributed. Hence, both positive and negative deviations would be equally likely.

When the estimand is the mean, and positive and negative deviations from the mean are penalised equally, then the squared error is a consistent loss function, or performance measure (25). Here consistency means that the performance measure is minimised when model estimates equal the estimand. The primary performance measure used was the mean (of the) squared error (MSE), with bias as a secondary performance measure. In addition to being a consistent loss function, the MSE has the benefit that it may be interpreted as penalising for both bias (how close are the model estimates to the truth) and variance (how much do estimates vary across simulations). Use of bias as a secondary measure provides insight into how the two components of bias and variance contribute to the MSE. The MSE and bias are defined as (16):

$$\text{MSE}_{i}=\frac{1}{n_{\text{sim}}}\sum_{j=1}^{n_{\text{sim}}} \left( \hat{\theta_{j,i}}-\theta_{i} \right)^{2}$$

$$\text{Bias}_{i}=\frac{1}{n_{\text{sim}}}\sum_{j=1}^{n_{\text{sim}}} \left( \hat{\theta_{j,i}}-\theta_{i} \right)$$

Where $n_{\text{sim}}$ is the number of simulations, $\theta_{i}$ is the estimand, and $\hat{\theta}_{j,i}$ is the corresponding model-based estimate (subscripts i, j denote time and simulation). For MSE lower values indicate better model performance, for bias values closer to zero indicate better model performance. As the hazard function is a time-varying estimand the performance measures are also time-varying. Summary (mean) values of the MSE and bias were calculated separately for the out-of-sample (extrapolations) and within-sample time periods. These summary measures use a novel method to calculate; the values of interest are:

$$f(x)=\frac{1}{T}\sum_{i=1}^{T} \mu_{i}$$

Where $\mu_{i}$ is the MSE or bias (with corresponding standard deviation of $\sigma_{i}$). Whilst $E\left[ f(x) \right]$ is a weighted mean, there is no standard expression to calculate $\text{Var}\left[ f(x) \right]$. Here a simulation-based approach is used. It is assumed that the MSE $\sim Gamma\left( \frac{\mu_{i}^{2}}{\sigma_{i}^{2}},\frac{\sigma_{i}^{2}}{\mu_{i}} \right)$ and the bias $\sim N\left( \mu_{i},\sigma_{i}^{2} \right)$. MSE and bias values were sampled 1,000 times from these distributions and used to generate percentile-based confidence intervals.

The time-horizon was 20 years (after which survival was essentially 0%), with time-steps of 0.05 years.

## Case study methods: further details

The data used were from the international COU-AA-301 trial. Patients were randomised 2:1 to receive either abiraterone or placebo, both study groups also received prednisone. Treatment was until disease progression and the primary outcome was overall survival. In total 1,195 patients were enrolled (abiraterone = 797, placebo = 398). A pre-specified interim analysis was published in 2011, based on an early data cut of 552 deaths (planned deaths = 534), with a median follow-up of 12.8 months (21). A further analysis was published after 775 deaths occurred (26). Following this analysis, the study was un-blinded and the remaining 18 patients in the placebo group (5%) crossed-over to receive abiraterone. An adjustment for treatment switching to abiraterone is not considered here due to the small numbers affected.

The data used in this study were made available by Janssen Research and Development, L.L.C. via the Yale University Open Data Access Project and represent more complete follow-up than either of the previous analyses, with 984 deaths (82.3% of the original study population). Data are for individual patients and were accessed via a secure online platform. For these data survival times (deaths or censorings) are measured as times from randomisation. As calendar times (for study entry) are not available, the early data cut was replicated based on the following steps:

1. Find the follow-up time of the 552^nd^ death in the dataset. Let this time = $T*$.
2. Censor all follow-up times greater than $T*$
3. For newly censored times, set the follow-up time = time of last study visit.

One further difference from the original interim analysis is that 12 people did not have any event or censoring times, so were excluded from all analyses (hence N = 1,195 – 12 = 1,183).

A comparison of the available data and the published early data cut is provided in Table A1. The replicated and published early cuts are generally very similar, albeit with a slight under-estimate of median survival for the replicated abiraterone group. Median follow-up in the full dataset is almost three times that of the early cut, with almost twice as many deaths. Of those who had not died in the full dataset, 155 (13.1% of the total sample) were still alive (and so censored at the last study visit day), with a further 38 patients withdrawn from the study and 6 lost to follow-up (and so censored at the recorded timings of the study-events).

**Table A1: available data and comparison with published early data cut.**

|  | **Published early data cut** | **Data used in this case-study** | |
| --- | --- | --- | --- |
|  |  | **Early cut replicated** | **Full dataset** |
| Sample size: Abiraterone, Placebo | 797, 398 | 791, 392 | 791, 392 |
| Median follow-up | 12.8 | 13.1 | 36.2 |
| Median survival: |  |  |  |
| Abiraterone | 14.8 | 13.9 | 15.9 |
| Placebo | 10.9 | 11.1 | 11.2 |
| Deaths: Total  (Abiraterone, Placebo) | 552 (46.2%)  (333, 219) | 552 (46.7%)  (329, 223) | 984 (83.2%)  (651, 333) |

## Simulation study: plots for additional results

**Figure A2: Model estimates of the log-hazard (blue lines) and true values (black lines), sample size = 100.**


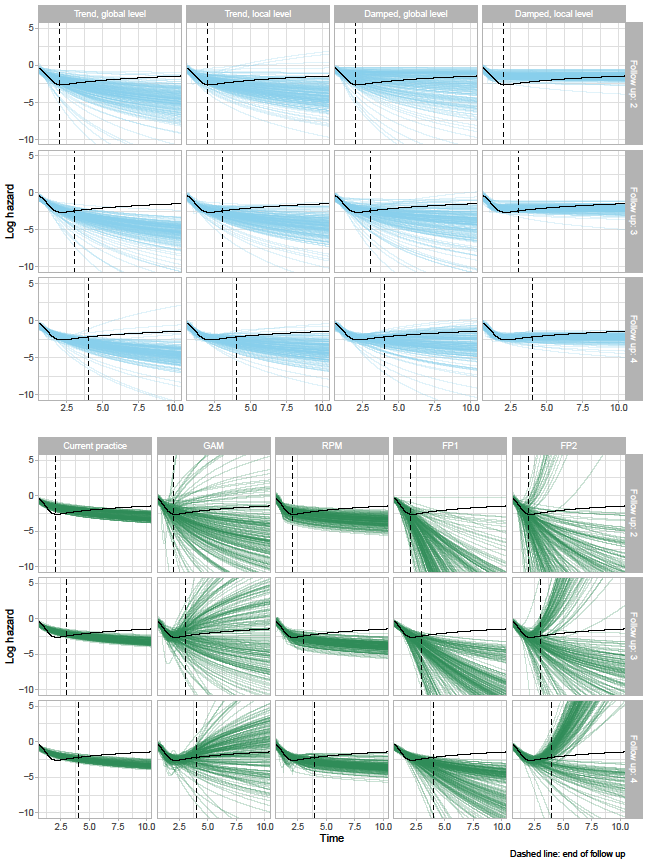


**Figure A3: Model estimates of the log-hazard (blue lines) and true values (black lines), sample size = 600.**


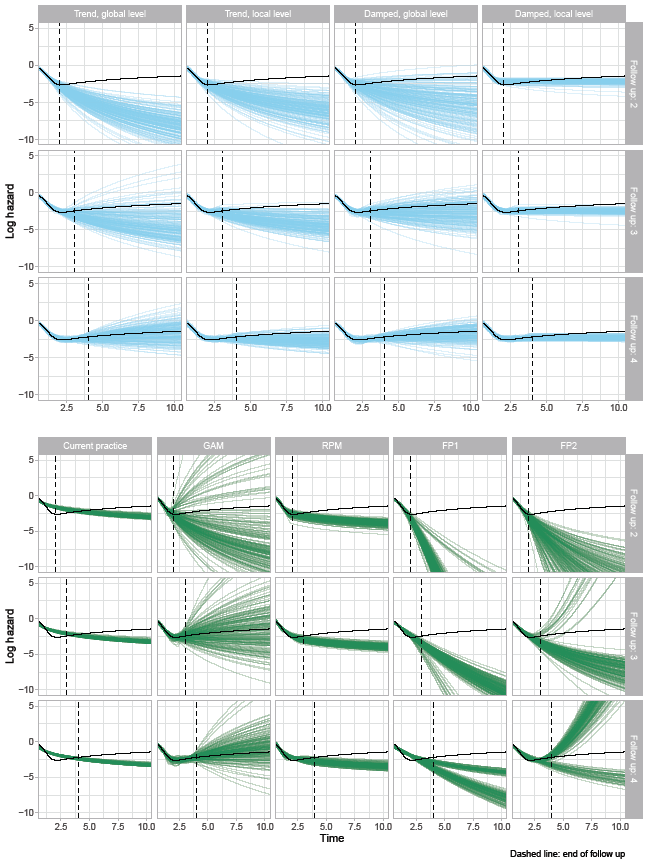


**Figure A4: Mean squared error and bias values by time (within-sample and extrapolations), sample size = 100.**


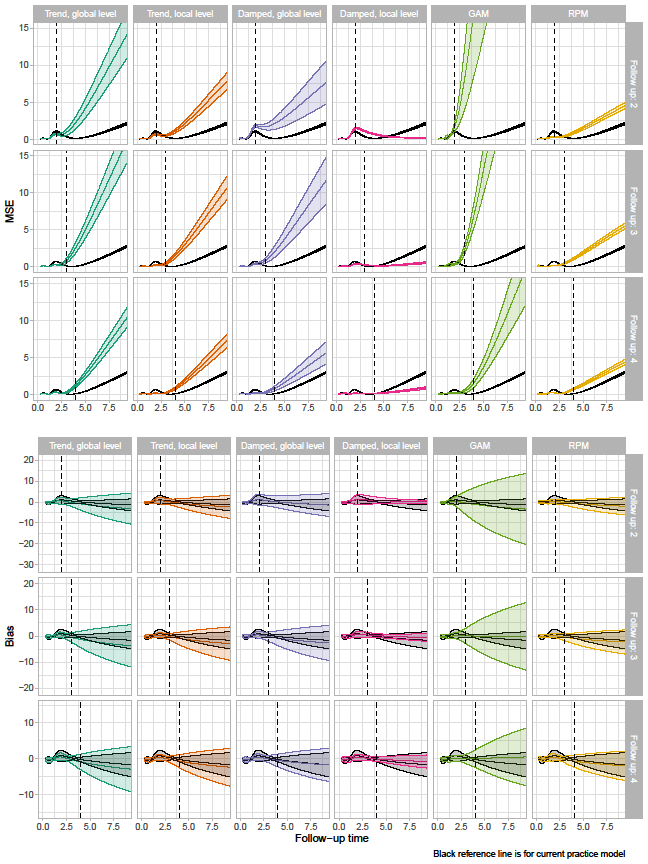


**Figure A5: Mean squared error and bias values by time (within-sample and extrapolations), sample size = 600.**


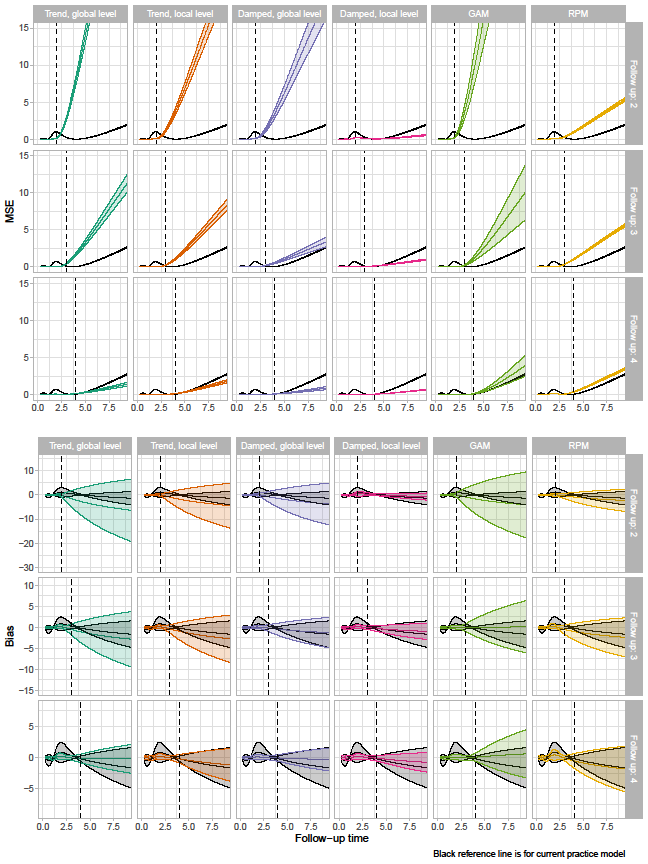


## Current practice resulting including the Gompertz model

Amongst the seven current practice models considered, the Gompertz provided both some of the best within-sample estimates and some of the worst extrapolations. Hence including it in the main analysis would have resulted in drastically decreased extrapolation performance for the current practice models. To illustrate this, the goodness of fit of each of the seven current practice models is visualised in Figure A6 for the three scenarios with a sample size of 300. No model captures the long-term increasing hazards, with all models extrapolating a decreasing trend. Of the seven models, the Gompertz is the only one that does not model time on the logarithm scale but uses the original time-scale (5). Use of the logarithm of time compresses the time scale. For example, the difference between two- and ten-years follow-up decreases to 1.6 years on the logarithm scale. Hence, models that use the logarithm scale for time can lead to less extreme extrapolations than those from the Gompertz model.

Summary MSE values are provided in Table A2, separately for the observed and extrapolated time periods. Based on within-sample fit, the Gompertz had the lowest average MSE in six of the scenarios, and the second lowest MSE (behind the generalised gamma) in the remaining scenarios. The Gompertz always had the largest out-of-sample MSE, with values that were always at least 16 times larger than the next worst extrapolating model (and more than 200 times worse than the best extrapolating current practice model in five of the scenarios). Results for bias were similar: the Gompertz had the best within-sample and worse out-of-sample values for all nine scenarios. The best extrapolating models based on MSE (bias) were the Weibull (lognormal), gamma (Weibull), and gamma (gamma) for follow-ups of two, three, and four years respectively (irrespective of sample size). Of note, the generalised gamma nearly always provided worse extrapolations on average than its special cases (the gamma, Weibull, and lognormal). This suggests that the additional flexibility of the generalised gamma led to over-fitting temporary trends in the data. Based on the individual simulation results, the Gompertz and generalised gamma models had the lowest within-sample MSE in 66% and 34% of simulations, respectively (no other model ever had the lowest within-sample MSE). The Gompertz never gave the best extrapolations, and the generalised gamma was only the best extrapolating model in 0.2% of simulations (4 / 1,800). The gamma was most frequently the best extrapolating model (55% of simulations), followed by the Weibull (30%) and Lognormal (12%). Whilst the Gompertz model always provided poor extrapolations for this simulation study, it may be a useful model in other circumstances.

**Figure A6: Model estimates of the log-hazard (blue lines) and true values (black lines) for current practice models**


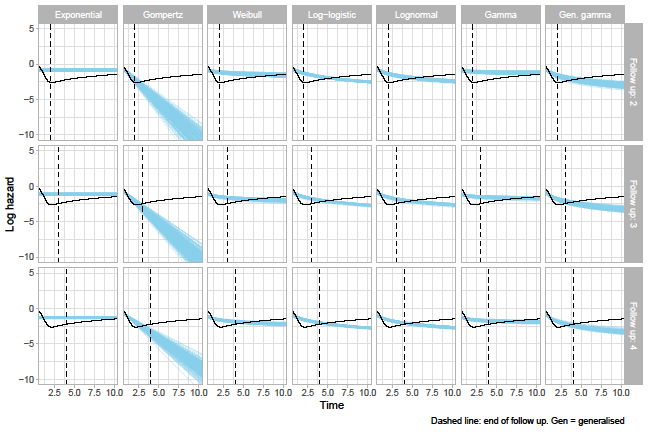


**Table A2: Summary within and out-of-sample mean-squared errors across scenarios (current practice models).**

|  | **Sample size = 100** | | **Sample size = 300** | | **Sample size = 600** | |
| --- | --- | --- | --- | --- | --- | --- |
| **Average MSE values** | **In sample** | **Out of sample** | **In sample** | **Out of sample** | **In sample** | **Out of sample** |
| **Follow-up = 2 years** |  |  |  |  |  |  |
| Exponential | 0.90 | 1.41 | 0.88 | 1.39 | 0.88 | 1.40 |
| Gompertz | 0.16 | 47.29 | 0.12 | 46.27 | 0.11 | 45.82 |
| Weibull | 0.63 | 0.66 | 0.59 | 0.59 | 0.59 | 0.59 |
| Log-logistic | 0.53 | 0.77 | 0.49 | 0.75 | 0.49 | 0.75 |
| Lognormal | 0.46 | 0.67 | 0.43 | 0.66 | 0.43 | 0.65 |
| Gamma | 0.70 | 0.86 | 0.66 | 0.79 | 0.66 | 0.80 |
| Generalised gamma | 0.29 | 1.33 | 0.29 | 1.18 | 0.29 | 1.12 |
| **Follow-up = 3 years** |  |  |  |  |  |  |
| Exponential | 1.25 | 0.73 | 1.20 | 0.67 | 1.21 | 0.68 |
| Gompertz | 0.18 | 49.02 | 0.15 | 46.96 | 0.14 | 47.32 |
| Weibull | 0.69 | 0.31 | 0.65 | 0.28 | 0.65 | 0.27 |
| Log-logistic | 0.61 | 0.78 | 0.57 | 0.78 | 0.57 | 0.78 |
| Lognormal | 0.54 | 0.71 | 0.50 | 0.71 | 0.50 | 0.71 |
| Gamma | 0.79 | 0.28 | 0.74 | 0.24 | 0.75 | 0.23 |
| Generalised gamma | 0.29 | 1.75 | 0.29 | 1.66 | 0.29 | 1.60 |
| **Follow-up = 4 years** |  |  |  |  |  |  |
| Exponential | 1.11 | 0.35 | 1.09 | 0.33 | 1.09 | 0.33 |
| Gompertz | 0.38 | 36.20 | 0.32 | 33.41 | 0.31 | 33.57 |
| Weibull | 0.53 | 0.36 | 0.51 | 0.33 | 0.51 | 0.33 |
| Log-logistic | 0.47 | 0.98 | 0.45 | 0.97 | 0.44 | 0.97 |
| Lognormal | 0.42 | 0.91 | 0.40 | 0.90 | 0.40 | 0.90 |
| Gamma | 0.61 | 0.19 | 0.59 | 0.16 | 0.59 | 0.15 |
| Generalised gamma | 0.22 | 2.24 | 0.22 | 2.07 | 0.22 | 2.00 |

## Comparison of spline-based models.

For both RPMs and GAMs, model flexibility is determined by the complexity of the model: for RPMs this is the number of internal knots (varying between 0 and 5 inclusive), which was based on minimising AIC. For GAM model complexity is based on the number of parameters; as these are shrunk during parameter estimation, the result is a non-integer number (between 0 and 10 inclusive). Results for RPMs and GAMs are provided in Figure A7 and Table A3, respectively. Of note, the RPMs never chose a model with zero internal knots (which would be equivalent to a Weibull model). For the shortest available sample size, there was a preference for simple one-knot models. For the remaining sample sizes, as follow-up increased there was an increased tendency to select more complex models. For GAMs there was a strong tendency for more complex models in the scenarios with increasing sample size or follow-up. Given that the extrapolation performance of GAMs increased in these scenarios, this suggest that increasing data richness allows for models of increasing complexity. The total number of parameters in the RPMs is the number of internal knots plus two. Hence, the RPMs had a tendency to select less complex models than the GAMs. For example, with a sample size of 600 and follow-up of 3 years, most RPMs typically had four parameters, whilst the GAMs had on average 6.67 parameters.

**Figure A7: Number of internal knots chosen for Royston-Parmar models.**


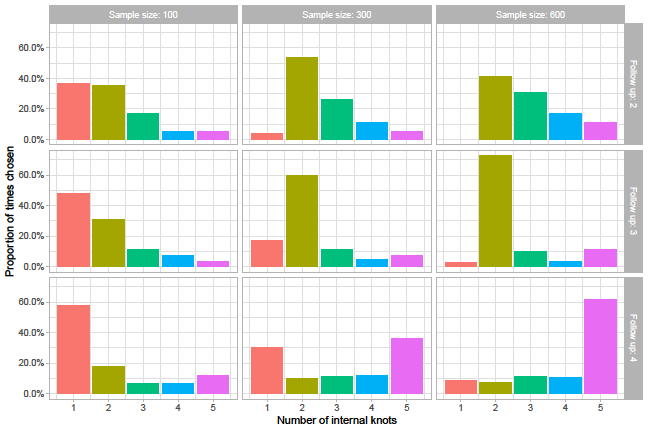


**Table A3: Model complexity: generalised additive models**

| **Sample size** | **Follow up** | **Mean** | **95% confidence interval** |
| --- | --- | --- | --- |
| 100 | 2 years | 4.06 | 2.00 to 7.92 |
| 100 | 3 years | 5.28 | 2.00 to 8.78 |
| 100 | 4 years | 5.51 | 2.00 to 9.08 |
| 300 | 2 years | 5.11 | 3.81 to 7.96 |
| 300 | 3 years | 6.35 | 4.35 to 9.54 |
| 300 | 4 years | 6.67 | 5.36 to 9.47 |
| 600 | 2 years | 5.95 | 4.30 to 9.37 |
| 600 | 3 years | 6.99 | 5.31 to 9.81 |
| 600 | 4 years | 7.36 | 6.04 to 9.77 |

## Definition of the inverse evidence ratio

Whilst models with lower information criteria values are preferred, it can be hard to evaluate meaningful differences in these values, as there are no guidelines for non-nested models. To alleviate this, the inverse evidence ratio (IER) may be used. This is a measure of how plausible a model is, relative to the ‘best’ model (the model with the minimum information criteria). Let ${IC}_{m}$ be the information criteria value (such as Akaike information criteria) for model $m$, and $IC*$ be the minimum of ${IC}_{m}$. Then the IER for model $m$ is defined as: $\exp\left( -0.5*\left[ IC_{m}-IC^{*} \right] \right)$. The best fitting model will always have an ${IC}_{m}=100\%$, whilst values for poorly fitting models will be close to zero (27). IER values are calculated within each class of model.

## Case-study: additional results

**Table A4: Case-study: goodness of fit measures**

|  | **Abiraterone** | | | | **Placebo** | | | |
| --- | --- | --- | --- | --- | --- | --- | --- | --- |
| **Current practice models** | **AIC** | **IER** | **BIC** | **IER** | **AIC** | **IER** | **BIC** | **IER** |
| Weibull | 1,086.3 | 100% | 1,093.9 | 100% | 536.3 | 87% | 543.1 | 87% |
| Gamma | 1,086.6 | 85% | 1,094.2 | 85% | 536.0 | 100% | 542.8 | 100% |
| Log-logistic | 1,088.2 | 39% | 1,095.8 | 39% | 539.6 | 16% | 546.4 | 16% |
| Generalised gamma | 1,088.3 | 37% | 1,099.6 | 6% | 538.0 | 37% | 548.2 | 7% |
| Generalised F | 1,090.3 | 14% | 1,105.4 | 0% | 540.0 | 14% | 553.6 | 0% |
| Gompertz | 1,096.0 | 1% | 1,103.5 | 1% | 545.1 | 1% | 551.9 | 1% |
| Lognormal | 1,102.6 | 0% | 1,110.2 | 0% | 542.5 | 4% | 549.3 | 4% |
| Exponential | 1,135.6 | 0% | 1,139.4 | 0% | 580.8 | 0% | 584.2 | 0% |
| **Royston-Parmar models** | **AIC** | **IER** | **BIC** | **IER** | **AIC** | **IER** | **BIC** | **IER** |
| Hazard scale; 0 knots | 1,086.3 | 100% | 1,093.9 | 100% | 536.3 | 5% | 543.1 | 100% |
| Hazard scale; 1 knots | 1,088.2 | 39% | 1,099.6 | 6% | 537.9 | 2% | 548.2 | 7.99% |
| Hazard scale; 2 knots | 1,090.2 | 14% | 1,105.4 | 0% | 537.4 | 3% | 551.0 | 2% |
| Hazard scale; 3 knots | 1,091.9 | 6% | 1,110.9 | 0% | 533.0 | 28% | 550.0 | 3% |
| Hazard scale; 4 knots | 1,092.8 | 4% | 1,115.6 | 0% | 535.1 | 10% | 555.6 | 0% |
| Hazard scale; 5 knots | 1,093.7 | 3% | 1,120.2 | 0% | 530.4 | 100% | 554.3 | 0% |
| Odds scale; 0 knots | 1,088.2 | 39% | 1,095.8 | 39% | 539.6 | 1% | 546.4 | 19% |
| Odds scale; 1 knots | 1,089.2 | 23% | 1,100.6 | 3% | 540.7 | 1% | 550.9 | 2% |
| Odds scale; 2 knots | 1,090.8 | 10% | 1,106.0 | 0% | 537.1 | 4% | 550.8 | 2% |
| Odds scale; 3 knots | 1,092.4 | 5% | 1,111.4 | 0% | 533.5 | 21% | 550.6 | 2% |
| Odds scale; 4 knots | 1,092.8 | 4% | 1,115.6 | 0% | 536.2 | 6% | 556.6 | 0% |
| Odds scale; 5 knots | 1,093.8 | 2% | 1,120.3 | 0% | 530.9 | 80% | 554.7 | 0% |
