# Supplementary material for: Comparing current and emerging practice models for the extrapolation of survival data: a simulation study and case-study
Source: BMC Med Res Methodol. 2021 Nov 27;21:263. doi: 10.1186/s12874-021-01460-1 (PMC8627632; doi:10.1186/s12874-021-01460-1)
Supplement: Supplementary file 2 — Additional file 2. [file 12874_2021_1460_MOESM2_ESM.docx]

# Appendix 2: Code used in analyses

Below is the R code used to perform the simulation study. For conciseness, code used to generate graphs has been omitted.

## Prepare data

library("here")

library("tidyverse")

library("survival")

library("gridExtra")

theme_set(theme_light())

here()

large_num = 99

# Define input parameters

my_g1 = 1.8

my_g2 = 1.4

my_l1 = 0.02

my_l2 = 2.3

my_time = seq(from=0.25, to=20, by=0.05) # For 'true' values (hazard not always defined at t=0)

# Replicate 2-component Weibull mixture from survsim https://www.stata-journal.com/article.html?article=st0275

fun_stats =function(x, p, l1, g1, l2, g2){

cum_surv = p * exp(-l1*(x^g1)) + (1-p) * exp(-l2*(x^g2))

pdf_surv = l1*g1*(x^(g1-1)) * p * exp(-l1*(x^g1)) + l2*g2*(x^(g2-1)) * (1-p) * exp(-l2*(x^g2))

cum_haz = (-1)*log(cum_surv)

haz = pdf_surv / cum_surv

my_out=list(

cum_surv = cum_surv,

pdf_surv = pdf_surv,

cum_haz = cum_haz,

haz = haz

)

return(my_out)

}

# Function to create data-frame with surival, hazard etc.

df_fun = function(my_p, my_row, my_from, my_to, my_by, l1, g1, l2, g2){

# Passed-arguments = my_row, my_from, my_to, my_by, l1, g1, l2, g2

my_df = data.frame(matrix(NA, nrow = my_row, ncol = 4))

names(my_df) = c("time", "cum_surv","pdf_surv","haz")

my_ind = 0

for(x in seq(from=my_from, to=my_to, by=my_by)){

my_ind = my_ind +1

tmp = fun_stats(x, p=my_p, l1=l1, g1=g1, l2=l2, g2=g2)

my_df$time[my_ind] = x

my_df$cum_surv[my_ind] = tmp$cum_surv

my_df$pdf_surv[my_ind] = tmp$pdf_surv

my_df$cum_haz[my_ind] = tmp$cum_haz

my_df$haz[my_ind] = tmp$haz

}

# Estimates of hazard very volatile when survival is small - set to 0 in this case (also adjust cumulative hazard).

my_df$haz = case_when(my_df$cum_surv < 0.001 ~ NaN, TRUE ~ my_df$haz)

my_df$cum_haz = case_when(my_df$cum_surv < 0.001 ~ NaN, TRUE ~ my_df$cum_haz)

# Derive conditional probability from conditional hazard.

my_df$prob = 1-exp(-my_df$haz*(my_df$time - lag(my_df$time, default=0)))

my_df$Mix = my_p # Save input value

return(my_df)

}

my_p = list(0, 0.5, 1)

my_df = map_dfr(my_p, df_fun, my_row=2000, my_from=0.02, my_to=40,

my_by=0.02, l1=my_l1, g1=my_g1, l2=my_l2, g2=my_g2)

########################################

## SIMULATION CODE ##

########################################

# Now to simulate survival times

n = 1000 # Observations

my_p = 0.5

sims = 200 # Number of simulations

set.seed(1616) # Dataset of random numbers

df_rnds = matrix(runif(n*sims), nrow=n, ncol=sims)

# Dataset of survival times: derived from the mixture Weibull model (see Excel file for values)

# Cannot use fun_stats directly in below, so define new function

fun_weib_mix = function(x, p, l1, g1, l2, g2, rnd=0){

surv = p * exp(-l1*(x^g1)) + (1-p) * exp(-l2*(x^g2))

return(surv - rnd)

}

df_surv = matrix(0, nrow=n, ncol=sims)

for(k in 1:sims){

for(i in 1:n){

df_surv[i,k] = tryCatch(uniroot(f = fun_weib_mix, p=my_p, l1=my_l1, g1=my_g1, l2=my_l2, g2=my_g2, rnd=df_rnds[i,k],

interval = c(0,large_num), extendInt="downX")$root, error = function(e) large_num)

# Above: sometimes get error if true survival > large_num (so set = large_num)

}

}

df_surv = data.frame(df_surv)

# Now get true values of the hazard for each year.

df_tru = data.frame(Time=my_time, Haz_0.5=fun_stats(x=my_time, p=0.5, l1=my_l1, g1=my_g1, l2=my_l2, g2=my_g2)$haz,

Surv_0.5=fun_stats(x=my_time, p=0.5, l1=my_l1, g1=my_g1, l2=my_l2, g2=my_g2)$cum_surv)

########################################

## Generate scenarios ##

########################################

fun_scens = function(my_n, study_FU, my_df, name){

# First get correct sample size

my_df = data.frame(df_surv[,my_df])

my_df = sample_frac(my_df, my_n/dim(my_df)[1], replace=FALSE)

# Generate time-on-study (in abscence of death)

colnames(my_df)[1] = "Tru_surv"

my_df$Follow_Up = study_FU

my_df = my_df %>% mutate(Censor = case_when(Tru_surv < Follow_Up ~ 0,

TRUE ~ 1),

Obs_surv = case_when(Censor==1 ~ Follow_Up,

TRUE ~ Tru_surv),

Scenario = name)

return(my_df)

}

for(i in 1:sims){

message(paste(i,""),appendLF=FALSE)

scenarios = expand.grid(my_n = c(100, 300, 600), study_FU = c(1, 2, 3, 4), my_df = i)

scenarios$name = paste0("df_S", rownames(scenarios))

if(i == 1){

scens = pmap_dfr(scenarios, fun_scens) %>% mutate("Sim" = i)

} else {

tmp = pmap_dfr(scenarios, fun_scens) %>% mutate("Sim" = i)

scens = bind_rows(scens, tmp)

}

}

df_full = scens %>% group_by(Scenario, Sim) %>% nest(.key="IPD")

# Above is IPD for scenarios. Also want to aggregate to time-points used in 'true' data

fun_agg = function(x){

tmp_time = arrange(subset(x, Censor==0), Obs_surv)$Obs_surv

tmp = mutate(x, Index = findInterval(x$Obs_surv, vec=tmp_time)) %>% # Gets intervals for events

group_by(Index) %>% # Per interval, summary stats

summarise(Count = n(),

Censor = sum(Censor),

Events = Count - Censor)

tmp = tmp %>% mutate(Time = tmp_time[Index], # New variables

Alive = sum(Count) - cumsum(lag(Count, default=0)),

Tau = lead(Time) - Time,

Ln_Tau = lead(log(Time)) - log(Time),

AtRisk = Alive * Tau,

haz_t = Events / AtRisk,

p_t = 1 - exp(-haz_t * (lead(Time) - Time))) %>%

filter(Time > 0) %>% na.omit %>% select(-Index) # Remove t = 0 for mods using log-time, remove rows with no data

# Remove t = 0 (models which use log-time cannot estimate this), add truth.

return(tmp)

}

df_full = df_full %>% mutate(Agg = map(IPD, fun_agg),

Truth = list(select(df_tru, -Time)))

df_full$Scenario = as.numeric(str_sub(df_full$Scenario, 5, -1L))

# Next add 'true' values for hazarrd, sample size (survival) and prob. This depends on scenario

tmp_fun = function(Scenario, dat){

dat = dat %>% mutate(Haz_tru = Haz_0.5,

Sam_tru = Surv_0.5,

Prb_tru = 1 - exp(-Haz_tru)) %>%

select(-c(Haz_0.5, Surv_0.5)) %>% mutate(Time = my_time)

return(dat)

}

df_full = df_full %>% mutate(Truth = map2(Scenario, Truth, tmp_fun)) # Note over-writing Truth

# Meta data on scenarios (mix already previously defined)

scen_meta = expand.grid(Sample = c(100, 300, 600),

Follow = c(1, 2, 3, 4),

Mixing = 0.5) %>% rowid_to_column("Scenario")

df_full = left_join(df_full, scen_meta, by = "Scenario")

#------------------------------------------------------------------------

# Save the data

saveRDS(df_full, here("Output", "Fullv2.rds")) # IPD and aggregaged data (latter also includes truth)

#------------------------------------------------------------------------

## Fit non-dynamic models

library("tidyverse")

library("flexsurv")

library("mgcv")

library("here")

set.seed(81466)

part = "_p4.rds" # Used for if the input is 'chopped' into parts (done separately)

small_num = 1*10^-9 # As sometimes everyone is dead

my_df = readRDS(here("Output", paste0("df_full", part)))

my_time = seq(from=0.25, to=20, by=0.05) # Time values for which we want estimates.

new_df = tibble(Time = my_time, Time2 = log1p(Time), AtRisk = 1)

num_scen = length(unique(my_df$Scenario)) # Num scenarios

my_dists = list("exp","weibull","gamma","lnorm","llogis","gengamma")

mod_TSD = function(df){ # Fit standard models - NB didn't always work for Gen. F. so excluding

best = flexsurvreg(Surv(Obs_surv, 1-Censor) ~ 1, data = df, dist = my_dists[[6]])

my_res = summary(best, t=my_time, type="hazard")

my_aic = best$AIC

my_name = my_dists[[6]]

for(i in 1:5){

tmp = flexsurvreg(Surv(Obs_surv, 1-Censor) ~ 1, data = df, dist = my_dists[[i]])

if(tmp$AIC < best$AIC){

best = tmp

my_res = summary(best, t=my_time, type="hazard")

my_aic = best$AIC

my_name = my_dists[[i]]

}

}

df = flatten(my_res)

df = tibble(Time = my_time, Pred = df$est, Low = df$lcl, Upp = df$ucl)

lst = list(df = df, AIC = my_aic, Comp = my_name)

return(lst)

}

mod_RP = function(df, max_k, my_scale){

best = flexsurvspline(Surv(Obs_surv, 1-Censor) ~ 1, data = df, k = 0, scale = my_scale)

my_res = summary(best, t=my_time, type="hazard")

my_aic = best$AIC

my_k = 0

for(i in 1:max_k){

tmp = flexsurvspline(Surv(Obs_surv, 1-Censor) ~ 1, data = df, k = i, scale = my_scale)

if(tmp$AIC < best$AIC){

best = tmp

my_res = summary(best, t=my_time, type="hazard")

my_aic = best$AIC

my_k = i

}

}

df = flatten(my_res)

df = tibble(Time = my_time, Pred = df$est, Low = df$lcl, Upp = df$ucl, Model = "RP")

lst = list(df = df, AIC = my_aic, Comp = my_k)

return(lst)

}

mod_GAM = function(df){ # Fit GAMs - nb default for k = 10

df$AtRisk = case_when(df$AtRisk == 0 ~ small_num, TRUE ~ df$AtRisk)

best = gam(Events ~ s(log1p(Time)) + offset(log(AtRisk)), data=df, family=poisson(link="log"))

my_res = predict(object=best, newdata=new_df, type="link", se.fit=TRUE)

df = tibble(Time = my_time, Pred=exp(my_res$`fit`),

Low = exp(my_res$`fit` + qnorm(0.025) * my_res$se.fit),

Upp = exp(my_res$`fit` + qnorm(1 - 0.025) * my_res$se.fit))

lst = list(df = df, AIC = best$aic, Comp = sum(best$edf1))

return(lst)

}

my_pow = c(-2,-1,-0.5,0.5,1,2,3)

mod_FP1L = function(df){

df$AtRisk = case_when(df$AtRisk == 0 ~ small_num, TRUE ~ df$AtRisk)

df$Time2 = log1p(df$Time)

# Start with log-transform

best = glm(Events ~ log(Time2) + offset(log(AtRisk)), data=df, family=poisson(link="log"))

my_res = predict(object=best, newdata=new_df, type="link", se.fit=TRUE)

my_aic = best$aic

my_p = 0

for(i in 1:7){

tmp = glm(Events ~ I(Time2^my_pow[i]) + offset(log(AtRisk)), data=df, family=poisson(link="log"))

if(tmp$aic < best$aic){

best = tmp

my_res = predict(object=best, newdata=new_df, type="link", se.fit=TRUE)

my_aic = best$aic

my_p = my_pow[i]

}

}

df = tibble(Time = my_time, Pred=exp(my_res$`fit`),

Low = exp(my_res$`fit` + qnorm(0.025) * my_res$se.fit),

Upp = exp(my_res$`fit` + qnorm(1 - 0.025) * my_res$se.fit))

lst = list(df = df, AIC = my_aic, Comp = my_p)

return(lst)

}

mod_FP2L = function(df){

df$AtRisk = case_when(df$AtRisk == 0 ~ small_num, TRUE ~ df$AtRisk)

df$Time2 = log1p(df$Time)

# Start with ones involving log; Log-log first

j = 1

best = glm(Events ~ log(Time2) + I(log(Time2)^2) + offset(log(AtRisk)), data=df, family=poisson(link="log"))

my_res = predict(object=best, newdata=new_df, type="link", se.fit=TRUE)

my_aic = best$aic

my_p = c(0,0)

# Now for the other logs

for (j in 1:7){

tmp = glm(Events ~ I(Time2^my_pow[j]) + I((Time2^my_pow[j])*log(Time2)) + offset(log(AtRisk)), data=df, family=poisson(link="log"))

if(tmp$aic < best$aic){

best = tmp

my_res = predict(object=best, newdata=new_df, type="link", se.fit=TRUE)

my_aic = best$aic

my_p = c(my_pow[j],my_pow[j])

}

tmp = glm(Events ~ I(log(Time2)) + I(Time2^my_pow[j]) + offset(log(AtRisk)), data=df, family=poisson(link="log"))

if(tmp$aic < best$aic){

best = tmp

my_res = predict(object=best, newdata=new_df, type="link", se.fit=TRUE)

my_aic = best$aic

my_p = c(0,my_pow[j])

}

}

# Now for everything else, k = FP1 power, j = FP2 power # WORKING ON THIS

for (k in 1:7){

for (j in 1:7){

if (j > k) {

tmp = glm(Events ~ I(Time2^my_pow[k]) + I(Time2^my_pow[j]) + offset(log(AtRisk)), data=df, family=poisson(link="log"))

if(tmp$aic < best$aic){

best = tmp

my_res = predict(object=best, newdata=new_df, type="link", se.fit=TRUE)

my_aic = best$aic

my_p = c(my_pow[k],my_pow[j])

}

}

}

}

df = tibble(Time = my_time, Pred=exp(my_res$`fit`),

Low = exp(my_res$`fit` + qnorm(0.025) * my_res$se.fit),

Upp = exp(my_res$`fit` + qnorm(1 - 0.025) * my_res$se.fit),

Model = "FP2")

lst = list(df = df, AIC = my_aic, Comp = my_p)

return(lst)

}

fun_LE = function(mod){ # Derive mean life-expectancy from model predictions

mod = mod %>% mutate(tau = Time - lag(Time, default=0),

cum_y = cumsum(Pred * tau),

surv = exp(-cum_y),

AUC = surv * tau)

LE = sum(mod$AUC)

return(LE)

}

fun_stats_all = function(mod, truth, FU){

df = left_join(mod, truth, by = "Time") %>% # Get truth, summary stats comparing truth and modelled ests

mutate(Bias = Pred - Haz_tru,

MSE = (Pred - Haz_tru)^2)

df_in = filter(df, Time <= FU)

df_out = filter(df, Time > FU)

Mean_Bias_in = weighted.mean(x=df_in$Bias, w=df_in$Sam_tru, na.rm=TRUE)

Mean_Bias_out = weighted.mean(x=df_out$Bias, w=df_out$Sam_tru, na.rm=TRUE)

Mean_MSE_in = weighted.mean(x=df_in$MSE, w=df_in$Sam_tru, na.rm=TRUE)

Mean_MSE_out = weighted.mean(x=df_out$MSE, w=df_out$Sam_tru, na.rm=TRUE)

my_out = list(Full = select(df, Bias, MSE, Time),

Mean_in = tibble(Mean_Bias_in, Mean_MSE_in),

Mean_out= tibble(Mean_Bias_out, Mean_MSE_out))

return(my_out)

}

fun_stats_mean = function(data){

Stats = pluck(data, "Mean_out")

}

#------------------------------------------------------------------------------------------------------

df_models = my_df %>% # Fit the models

mutate(mod_TSD = map(IPD, mod_TSD),

mod_RPM = map(IPD, mod_RP, max_k = 5, my_scale="hazard"),

mod_GAM = map(Agg, mod_GAM),

mod_F1L = map(Agg, mod_FP1L),

mod_F2L = map(Agg, mod_FP2L)) %>%

select(-c(IPD, Agg, Mixing)) %>%

gather(key="Model", value="Mod_ests", -c(Scenario, Sim, Follow, Sample, Truth))

df_models = df_models %>%

mutate(Mod_df = map(Mod_ests, pluck("df")),

AIC = map_dbl(Mod_ests, pluck("AIC")),

Complex = map(Mod_ests, pluck("Comp")),

LE_mod = map_dbl(Mod_df, fun_LE), # Model estimate of lifetime mean survival

stats_all = pmap(list(Mod_df, Truth, Follow), .f = fun_stats_all), # Matching inputs by position

stats_mean = map(stats_all, fun_stats_mean))

saveRDS(df_models, here("Output", "LogTime", paste0("df_models", part)))

## Fit dynamic models: example stan file

// The below code is for a local-level local-damped-trend model. This is the most general of models

// A global level is obtained by removing zeta1 (and the variables used to create this)

// A local level is obtained by removing references to phi

// In practice a seperate stan file is used for each model

data {

int<lower=1> T; // Time points

vector[T-1] tau; // Width between time-points

int y[T]; // Events

vector[T] n; // At risk

}

parameters {

real beta_01; // Initial coeff1

real<lower=0> Z1; // Variance coeff1

real<lower=0> Z2; // Variance coeff2

real beta_02; // Initial coeff2

vector[T] zeta_tilde1; // Tranformation of zeta2 (as in 8-schools example)

vector[T-1] zeta_tilde2; // Tranformation of zeta2 (as in 8-schools example)

real<lower=0.7, upper=0.999> phi; // Whether or not we know phi values (don't let = 1 as messes up extrap calcs)

}

transformed parameters {

vector[T] beta1; // State 1

vector[T-1] beta2; // State 2

{ // Don't want to save this

vector[T] zeta1; // Innovations

vector[T-1] zeta2; // Innovations

zeta1 = sqrt(Z1) * zeta_tilde1;

zeta2 = sqrt(Z2) * zeta_tilde2;

beta1[1] = beta_01 + zeta1[1];;

beta2[1] = beta_02 + zeta2[1];

for (t in 2:T-1) {

beta1[t] = beta1[t-1] + beta2[t-1] * phi * tau[t-1] + zeta1[t];

beta2[t] = beta2[t-1] * phi + zeta2[t];

}

beta1[T] = beta1[T-1] + beta2[T-1] * phi * tau[T-1] + zeta1[T];

}

}

model {

Z1 ~ inv_gamma(1, 0.005);

Z2 ~ inv_gamma(1, 0.005);

zeta_tilde1 ~ normal(0, 1);

zeta_tilde2 ~ normal(0, 1);

y ~ poisson(exp(beta1) .* n);

}

generated quantities{

real level;

real trend;

level = beta1[T];

trend = beta2[T-1];

}

## Fit dynamic models: example R code

library("tidyverse")

library("here")

library("rstan")

rstan_options(auto_write = TRUE)

# Below code is for fitting a local-level damped trend model. As noted in the stan file, some variations are required for fitting the other models, which would have their own files.

set.seed(81466)

part = "_p4.rds" # Used for if the input is 'chopped' into parts (done separately)

small_num = 1*10^-5 # As sometimes everyone is dead

my_df = readRDS(here("Output", paste0("df_full", part)))

max_h = 20

my_time = seq(from=0.25, to=max_h, by=0.05) # Time values for which we want estimates.

new_df = tibble(Time = my_time, AtRisk = 1)

num_scen = length(unique(my_df$Scenario)) # Num scenarios

my_counter = 0

mod_DSM = function(df){ # To add-in function for DSM models

df$AtRisk = case_when(df$AtRisk == 0 ~ small_num, TRUE ~ df$AtRisk)

df$Ln_Tau = log1p(df$Time) - lag(log1p(df$Time), default = 0)

my_counter <<- my_counter + 1

print(my_counter)

my_data1 <- list(

y = df$Events,

T = length(df$Events),

n = df$AtRisk,

tau = tail(df$Ln_Tau, -1)

)

init_list = list(list(beta_01 = log(min(1, my_data1$y[1] / my_data1$n[1])),

beta_02 = 0, Z = small_num)) # Z = small num

fit1 <- stan(

file = "DT_LL_IG.stan",

data = my_data1, # named list of data

chains = 1, # number of Markov chains

warmup = 1000, # number of warmup iterations per chain

iter = 2000, # total number of iterations per chain

cores = 1, # number of cores

init = init_list, # Initial values for DSM models

refresh = 0, # show progress every 'refresh' iterations

control = list(adapt_delta = 0.95, max_treedepth = 15)

)

# Within-sample estimates

beta1 = extract(fit1, pars = c("beta1"))

int_haz = map_dfr(beta1, function(x) colMeans(exp(x)))

tmp2 = tibble(Time = df$Time, mean = int_haz$beta1)

max_fu = max(tmp2$Time) # = tmp2$Time[length(tmp2$Time)] as ordered

time_int = filter(new_df, Time <= max_fu)

int_est = approx(x=tmp2$Time, y=tmp2$mean, xout=time_int$Time, rule=2)$y

# Extrapolations

# Get future tau (width) values for when to dampen trend - fit linear model to last half of data

df2 = mutate(df, Ind = row_number()) %>% filter(Ind > max(Ind)/2) %>% mutate(Ind = Ind - min(Ind) + 1)

mod_w = lm(Ln_Tau ~ Ind, data = df2)

tmp = data.frame(Ind = seq(from = max(df2$Ind), to = max(df2$Ind) + 1))

w_new = predict(mod_w, tmp) # Initital ests, to find out how long to make extrap widths.

new_upp = ceiling((max_h - max_fu) / (w_new[2] + max(mod_w$coefficients[2], 0)))

if (mod_w$coefficients[2] > 0) {

tmp = data.frame(Ind = seq(from = max(df2$Ind), to = max(df2$Ind) + new_upp + 1))

w_new = predict(mod_w, tmp)

} else {

w_new = rep(w_new[2], new_upp + 1)

}

w_df = tibble(time_new = cumsum(c(max_fu, w_new)))

# Estimates

tmp_df = tibble(Time = log1p(w_df$time_new) - log1p(max_fu)) %>% mutate(level = extract(fit1, pars = c("level")),

trend = extract(fit1, pars = c("trend")), phi = extract(fit1, pars = c("phi")),

ext_est = pmap(list(level, trend, phi, Time), function(level, trend, phi, Time) exp(level + trend * phi * (1 - phi^Time)/(1 - phi))),

Pred = map_dbl(ext_est, mean), Time = w_df$time_new,

Low = map_dbl(ext_est, function(x) quantile(x, probs = 0.025)),

Upp = map_dbl(ext_est, function(x) quantile(x, probs = 1 - 0.025)),

Phi_m = map_dbl(phi, mean), Trend_m = map_dbl(trend, mean), Level_m = map_dbl(level, mean)) %>%

select(Time, Pred, Low, Upp, Phi_m, Trend_m, Level_m)

fu_time = filter(new_df, Time > max_fu) %>% select(Time)

mod_est = tibble(Time = my_time,

Pred = c(int_est, approx(x = tmp_df$Time, y = tmp_df$Pred, xout = fu_time$Time)$y),

Low = c(int_est, approx(x = tmp_df$Time, y = tmp_df$Low, xout = fu_time$Time)$y),

Upp = c(int_est, approx(x = tmp_df$Time, y = tmp_df$Upp, xout = fu_time$Time)$y))

lst = list(df = mod_est, AIC = mean(tmp_df$Phi_m), Comp = mean(tmp_df$Trend_m), level = mean(tmp_df$Level_m))

return(lst)

}

#------------------------------------------------------------------------------------------------------

# Fit model in global environment once to avoid recompiling

my_data <- list(

y = rep(1,10),

T = 10,

n = seq(10,1,-1),

tau = rep(1,9)

)

temp_mod <- stan(

file = "DT_LL_IG.stan", # Stan program

data = my_data, # named list of data

chains = 1

)

df_models = my_df %>% # Fit the models

mutate(Local_level_damped_trend = map(Agg, mod_DSM)) %>%

select(-c(IPD, Agg, Mixing)) %>%

gather(key="Model", value="Mod_ests", -c(Scenario, Sim, Follow, Sample, Truth))

df_models = df_models %>%

mutate(Mod_df = map(Mod_ests, pluck("df")),

AIC = map_dbl(Mod_ests, pluck("AIC")),

Complex = map(Mod_ests, pluck("Comp")))

saveRDS(df_models, here("Output", "DSM_2020", paste0("df_models_DSM_DT_LL_Ln", part)))

## Generate results

library("tidyverse")

library("here")

library("gridExtra")

#--Part 1 Get data ready to generate results----------------------------------------------------------------

# Load data

df_files = vector(mode="list", length=1) # List to hold file names

df_files = list.files(path= here("Output", "LogTime"), pattern = "df_models_") # TSD select exc Gomp, GAM, FPs, RPM

df_modelsp1 = map_dfr(df_files, function(x) readRDS(here("Output", "LogTime", x)))

df_modelsp1$Model = fct_recode(df_modelsp1$Model, "Current practice" = "mod_TSD", "FP1" = "mod_F1L",

"FP2" = "mod_F2L", "GAM" = "mod_GAM", "RPM" = "mod_RPM")

df_files = list.files(path= here("Output", "IG_Prior", "V2"), pattern = "df_models_") # DSM (Ln time, global level)

df_modelsp2 = map_dfr(df_files, function(x) readRDS(here("Output", "IG_Prior", "V2", x)))

df_modelsp2$Model = fct_recode(df_modelsp2$Model, "Trend, global level" = "mod_DSM_Ln", "Damped, global level" = "mod_DSM_DT_Ln")

df_files = list.files(path= here("Output", "IG_Prior", "V2", "LL"), pattern = "df_models_") # DSM (Ln time, local level)

df_modelsp3 = map_dfr(df_files, function(x) readRDS(here("Output", "IG_Prior", "V2", "LL", x)))

df_modelsp3$Model = fct_recode(df_modelsp3$Model, "Trend, local level" = "Local_level_local_trend",

"Damped, local level" = "Local_level_damped_trend")

df_models = bind_rows(df_modelsp1, df_modelsp2, df_modelsp3)

rm(df_modelsp1, df_modelsp2, df_modelsp3)

gc()

df_tru= readRDS(here("Output", "Fullv2.rds"))

tru = df_tru %>% select(-c(IPD, Agg)) %>% unnest() %>% filter(Follow > 1) %>% mutate("Follow up" = Follow, "Sample size" = Sample)

fun_stats_all = function(mod, truth){

CI_a = 2/0.05 # For 95% CI - if change need to manually change here

df = left_join(mod, truth, by = "Time") %>% # Get truth, summary stats comparing truth and modelled ests

mutate(Err = log(Pred + small_n) - log(Haz_tru + small_n),

Err2 = (log(Pred + small_n) - log(Haz_tru + small_n))^2,

sMAPE = 2*abs(Err) / (abs(log(Pred + small_n)) + abs(log(Haz_tru + small_n))))

my_out = tibble(Time = df$Time, Err = df$Err, Err2 = df$Err2, sMAPE = df$sMAPE)

return(my_out)

} # Calculate error by time-point

table(df_models$Model)

df_models = select(df_models, -c("Mod_ests", "AIC", "Complex", "LE_mod", "stats_all", "stats_mean")) %>%

mutate(flag = map_dbl(Mod_df, function(x) if(max(abs(log(x$Pred[1:46])), na.rm=TRUE) > 10) 1 else 0)) %>%

filter(flag == 0)

gc()

table(df_models$Model) # Removed 84 FP1, 58 FP2, 2 GAM

df_ext = df_models %>% mutate(Mod_df = map2(Mod_df, Follow, function(x, y) filter(x, Time > y)),

Truth = map2(Truth, Follow, function(x, y) filter(x, Time > y)),

Err_time = map2(Mod_df, Truth, function(x, y) fun_stats_all(x, y)))

# Save one estimand per time-point

df_stats_full = df_ext %>% select(-c("Truth", "Mod_df")) %>% unnest(Err_time)

saveRDS(df_stats_full, here("Output", "Res_ext.rds"))

df_int = df_models %>% mutate(Mod_df = map2(Mod_df, Follow, function(x, y) filter(x, Time <= y)),

Truth = map2(Truth, Follow, function(x, y) filter(x, Time <= y)),

Err_time = map2(Mod_df, Truth, function(x, y) fun_stats_all(x, y)))

# Save one estimand per time-point

df_stats_full = df_int %>% select(-c("Truth", "Mod_df")) %>% unnest(Err_time)

saveRDS(df_stats_full, here("Output", "Res_int.rds"))

#--Part 2 Generate results----------------------------------------------------------------

library("tidyverse")

library("here")

library("gridExtra")

library("rsimsum")

df_tru= readRDS(here("Output", "Fullv2.rds")) # Truth

tru = df_tru %>% select(-c(IPD, Agg)) %>% unnest() %>% filter(Follow > 1 & Sim == 1) %>%

select(-c(Sim, Mixing, Haz_tru, Prb_tru)) %>% mutate("Follow up" = Follow, "Sample size" = Sample)

stats_int = readRDS(here("Output", "Res_int.rds")) %>% mutate(Period = "Int")

stats_ext = readRDS(here("Output", "Res_ext.rds")) %>% mutate(Period = "Ext")

stats_int$Model = fct_relevel(stats_int$Model, "Standard", "Trend v1", "Trend v2",

"Damped v1", "Damped v2", "GAM", "RPM", "FP1", "FP2")

stats_ext$Model = fct_relevel(stats_ext$Model, "Standard", "Trend v1", "Trend v2",

"Damped v1", "Damped v2", "GAM", "RPM", "FP1", "FP2")

stats_all = bind_rows(stats_int, stats_ext)

stats_all$Model = fct_recode(stats_all$Model, "Trend, global level" = "Trend v1", "Damped, global level" = "Damped v1",

"Damped, local level" = "Damped v2", "Trend, local level" = "Trend v2")

fun_stats = function(df_stats_full, int_ext){

# Calc MSE and bias per time-point

df = df_stats_full %>% na.omit %>% # 15 NAs for RPMs internal data

group_by(Scenario, Sample, Follow, Model, Time) %>%

summarise(MSE = mean(Err2), Bias = mean(Err), my_n = n(),

MSE_MC = (mean((Err2 - MSE)^2) / (my_n-1))^0.5,

Bias_MC = (MSE * my_n / (my_n-1))^0.5,

MSE_low = MSE - 1.96 * MSE_MC, MSE_upp = MSE + 1.96 * MSE_MC,

Bias_low = Bias - 1.96 * Bias_MC, Bias_upp = Bias + 1.96 * Bias_MC) %>%

mutate("Follow up" = Follow, "Sample size" = Sample)

if(int_ext == "Ext_") df$Time2 = df$Time - df$Follow else df$Time2 = df$Time

# Calculate MSE, Bias weighted over time-points (not pursuing sMAPE)

n_sim = 1000

df_new = left_join(df, tru, by = c("Scenario", "Sample size", "Follow up", "Time")) %>% ungroup() %>%

select("Scenario", "Sample size", "Follow up", "Time", "Model", "Sam_tru", "MSE", "Bias", "MSE_MC", "Bias_MC") %>%

mutate(MSE_new = map2(MSE, MSE_MC, function(x, y) rgamma(n_sim, shape = x^2 / y^2, rate = x / y^2)),

Bias_new = map2(Bias, Bias_MC, function(x, y) rnorm(n_sim, mean = x, sd = y))) %>%

unnest() %>% group_by(Time) %>% mutate(id = row_number()) %>% ungroup() %>%

group_by(Scenario, `Sample size`, `Follow up`, Model, id) %>% nest() %>% # This gies n_sim rows per model-scenario.

mutate(MSE_full = map_dbl(data, function(x) weighted.mean(x = x$MSE, w = x$Sam_tru, na.rm=TRUE)),

MSE_full2 = map_dbl(data, function(x) weighted.mean(x = x$MSE_new, w = x$Sam_tru, na.rm=TRUE)),

Bias_full = map_dbl(data, function(x) weighted.mean(x = x$Bias, w = x$Sam_tru, na.rm=TRUE)),

Bias_full2 = map_dbl(data, function(x) weighted.mean(x = x$Bias_new, w = x$Sam_tru, na.rm=TRUE))) %>%

group_by(Scenario, `Sample size`, `Follow up`, Model) %>%

summarise(MSE_full = mean(MSE_full), MSE_SE = sd(MSE_full2), MSE_full2 = mean(MSE_full2),

Bias_full = mean(Bias_full), Bias_SE = sd(Bias_full2), Bias_full2 = mean(Bias_full2),

MSE_low = MSE_full - 1.96 * MSE_SE, MSE_upp = MSE_full + 1.96 * MSE_SE,

Bias_low = Bias_full - 1.96 * Bias_SE, Bias_upp = Bias_full + 1.96 * Bias_SE) %>% ungroup()

saveRDS(df_new, here("Output", paste0(int_ext, "Res_full.rds")))

write.csv(df_new, here("Output", paste0(int_ext, "Res_full.csv")))

saveRDS(df_tmp, here("Output", paste0(int_ext, "Res_time.rds")))

write.csv(df_tmp, here("Output", paste0(int_ext, "Res_time.csv")))

}

fun_stats(stats_int, "Int_")

fun_stats(stats_ext, "Ext_")

fun_stats(stats_all, "All_")
